# Gynecology

**Published:** 1876-01

**Authors:** 


### II. Gynecology.

1. *Hypertrophy of the Vaginal Portion of the Cervix.* Dupuy. (*Le Progrès Médical*, Oct. 23.)

A primipara, aged 19, had, after delivery, hypertrophy of the cervix, whose projection beyond the vulva was as large as an apple. It was red and ulcerated, the ulcer being as large as a quarter of a dollar. Digital examination revealed a species of callous induration which marked the junction of the body and neck of the womb, the former being of normal size, while the latter was seven centimetres in length.

Under anæsthetics, the hypertrophied portion was removed in about a quarter of an hour, by the écraseur, and with the loss of but a few drops of blood. There was no obliteration of the cervical canal, though no measure, were taken to prevent it.

Dupuy refers to a similar operation performed by Warren, of Boston, (*Am. Jour. of Med. Sciences*), but considers the details given of the latter case "too scanty and too obscure to be available in a serious discussion of the subject." As is too common with many French writers, he exhibits a lack of the politeness which is supposed to be characteristic of his nation, by misspelling the name of the American surgeon.

2. *The Genu-Pectoral Posture and Pneumatic Pressure in the Treatment of Uterine Displacements.* Hopkins. (*Atl. Med. and Surg. Jour.*, Nov.)

The author having treated six cases successfully by the aid of Campbell's pneumatic self-repositor, is enthusiastic regarding its value. "Indebted solely to Dr. Campbell," he says, "for my success in the treatment of the cases here reported, it becomes my duty, while it affords me pleasure, to publicly acknowledge the same, and return him my sincere thanks for the valuable service rendered."

3. *Iodoform in Vaginismus.* Tarnier. (*Jour. de Méd. et de Chir.*, Sept.)

The hyperæsthesia was so extreme in a married woman, thirty-two years of age, married seventeen years, that walking was painful, coitus a torture, and the contact of a stylet with one of the labia minora elicited a cry of anguish.

Insensibility of the vulvar orifice was procured by dusting iodoform over the ostium vaginæ and lesser labia. The irritability disappeared for two days, but returned in a milder form. Then the dressing was repeated with a tampon, dusted over with iodoform, inserted between the labia. The results were exceedingly satisfactory, and Tarnier was hence led to recommend the same application in a case of fissure of the anus, which had been treated by astringents, narcotics and dilatation. The applications resulted in cure of the ailment at the expiration of ten months, relief being had much earlier.

4. *Hydrate of Chloral in Uterine Carcinoma.* FLEISCHER. (*Bulletin Général de Thér., Méd. et Chir.*)

The vagina having being cleansed by injections of water, cotton is applied to the carcinomatous surface, wet with a solution of chloral, eight parts to one hundred. The application is renewed every two hours, and, in two or three days, there is less pain and no fetidity of discharge. Administered by the rectum, its effects are completely under control and more prolonged; while it is less constipating than morphia.

5. *Rupture of Uterus—Recto-vaginal Fistula—Spontaneous Recovery.* FONTAINE. (*Virginia Medical Monthly*, Oct.)

A woman, 28 or 30 years of age, had contracted pelvis, and after many hours of labor, F. found the head had receded far up in the pelvic cavity. The breech was felt through the abdominal walls, with the feet and legs impinging upon the maternal liver. A rent had occurred in the right anterior quarter of the fundus uteri, which grasped the middle of the body of the fœtus. Craniotomy was immediately and rapidly performed, and extraction effected with pressure from above. Two-thirds of the placenta was removed from the abdominal cavity.

During the operation, fæces gushed into the vagina from a fistulous rent just below and a little to the left of the sacral promontory. It remained pervious, with passage of fœcal matter, for several weeks, but on the ninth week the speculum disclosed a rough puckered cicatrix, in the form of a raphé, one inch and a quarter long.

The cure was complete, after most skillful and judicious treatment—rest, carbolic acid injections, medicines to ensure solubility of the bowels, and a nutritious diet.

6. *Laceration of Perineum, Sphincter Ani and Recto-Vaginal Septum.* STERLING. (*Med. and Surg. Reporter*, Nov. 20.)

This accident having occurred, three deep-seated, double ligatures were passed with a curved needle, twenty-eight hours after delivery, the bowels being well evacuated. One was at the posterior fourchette, one at the verge of the anus, and a third midway between the two. Three superficial stitches were also taken. Opiates were administered to secure control of the bowels, but the escape of gas through the rent necessitated passing a silver tube into the rectum, which obviated the difficulty.

In four weeks the cure was complete, the operator taking the precaution to pack the vagina with sponge on the occurrence of the first stool, to avoid strain upon the new septum.

7. *Stricture of the Female Urethra.* NEWMAN. (*Am. Jour. of the Med. Sciences*, October, 1875.)

Of four cases, one occurred at the meatus ; another, three-fourths of an inch deeper ; still another, seven-eighths of an inch ; and a fourth case involved nearly the entire urethra. One was due to syphilis, one to irritating injections in gonorrhœa, one to granular u ethritis, and the last to general urethritis occupying the entire canal. These cases were relieved by electrolysis—positive pole in the palm, and a metallic bougie in the urethra, connected with the negative pole. From four to eleven cells of Drescher's battery were used. In one case of double stricture a "carneous plug" was expelled from the urethra, to the great relief of the patient.

8. *Chlorosis with Aplasia of the Female Genitals.* FRAENKEL. (*Archiv. für Gyn.*, vii.)

A woman of strong constitution, twenty-six years old, was chlorotic and had never menstruated. There was marked decrease in the number of red blood globules, but no heart lesion or disturbance.

Her breasts were infantile; her pelvis masculine and contracted in all diameters; the labia majora and hymen were wanting, while the nymphæ were imperfectly developed. The uterus was small, its parietes thin, and ovaries undistinguishable.

Another girl, twenty years old, had scarlatina and cholera in childhood, with first menstruation (profuse) at seventeen, accompanied by fainting, dyspnœa and palpitation. Pallor of the face and cold extremities were marked. There was apicial pneumonia with small heart; thin, thready pulse, with a weak impulse. The genitals were those of a girl seven or eight years old; mons without hair; small, infantile breasts and womb.

Fraenkel agrees with Virchow that chlorosis often accompanies genital aplasia, but not always defective development of the heart and aorta. It may result from the aplasia, but is permanently curable when the heart is not of small size. Menorrhagic chlorosis occurs with defective or excessive development of the sexual organs.

